# Adherence to Long-Term Therapies and Beliefs about Medications

**DOI:** 10.1155/2014/479596

**Published:** 2014-02-13

**Authors:** Abdullah AlHewiti

**Affiliations:** Department of Family Medicine and Primary Health Care, King Abdulaziz Medical City, National Guard Health Affairs, P.O. Box 22490, Riyadh 11426, Saudi Arabia

## Abstract

*Objectives*. To assess adherence to long-term medications among patients in family medicine clinics and to evaluate relationship between adherence, beliefs about medications, medication information adequacy, and other factors. *Methods*. Interviewer assisted survey was conducted to assess adherence using the 8-item Morisky Medication Adherence Scale (MMAS-8), beliefs about medications using beliefs about medicine questionnaire (BMQ), and the patients' perception of medication information adequacy. *Results*. Of the 408 participants, 56.9% reported low adherence. Pearson's bivariate correlation showed positive association between MMAS-8 score and BMQ-specific necessity (*r* = 0.526  *P* < 0.001) and the perceived information adequacy (*r* = 0.568  *P* < 0.001), and there was negative association between adherence score and BMQ specific concerns, general overuse, and harm (*r* = −0.647, −0.466, and −0.663, resp.) (*P* < 0.001). Multivariable analysis revealed that age, number of medications, number of medical conditions, specific necessity and concerns beliefs, general harm beliefs, and perceived adequacy of medication information were independent predictor of adherence. Furthermore, specific beliefs explain 27.7% of the variance in adherence, while medication information adequacy explains 32.3% of the variance in adherence. *Conclusion*. The prevalence of low adherence among patients on long-term medications is high and it is related to negative beliefs about medications and to inadequate information given to patients about their medications.

## 1. Introduction

According to clinical trials, many effective treatments exist for chronic diseases [1]. However the outcomes in the real world are not as expected from these explanatory trials and lack of adherence to treatment is considered one of the major reasons [1, 2]. It has been estimated that, in developed countries, patients with chronic medical illnesses adhere only 50% of the time to their medications, and it is believed that the problem is much higher in developing countries [2–4]. Furthermore, multiple efforts have been undertaken to understand nonadherence and many complex reasons and interventions have been proposed [2, 5]. However interventions designed to improve long-term adherence have not been effective by far, and an innovative way of looking at the problem is needed [6].

Generally nonadherence can be classified as either intentional or unintentional [7]. Intentional nonadherence as a behavior can be viewed in the context of theories of reasoned action and planned behavior, where individual's beliefs about certain behaviors are strong predictors of behavioral intention [8]. Therefore, holding positive beliefs about medications is a prerequisite for intentional adherence [9, 10]. Unintentional nonadherence such as forgetfulness, on the other hand, was considered to be due to regimen complexity; nevertheless, it has also been found to be influenced by beliefs [11]. There is a plethora of literature that supports the association between beliefs and adherence, and it has been proposed that beliefs are the strongest predictors of adherence [12, 13]. Furthermore, knowing beliefs is important, as they tend to be fixed over time unless an intervention is made [14]. An intervention such as education has been proven to change beliefs and there is evidence that targeting patients' specific beliefs improves adherence [15, 16].

Patients' background information and their knowledge about medications affect their beliefs about medications in different ways [15, 17]. Moreover, the impact of beliefs on adherence varies according to the culture [18, 19]. While beliefs about medications were studied in different parts of the world, little is known on how patients with chronic diseases in developing countries and in family medicine clinics view their medications and if their beliefs impact adherence. Furthermore, we do not know how our patients view the adequacy of the information given to them about their medications and how this could influence their beliefs and adherence. The aim of this study is to estimate adherence to long-term therapies among patients with chronic diseases and to examine the relationship between adherence, beliefs about medications, and patients' perception about the adequacy of medication information.

## 2. Materials and Methods

A cross-sectional interviewer assisted survey was conducted on patients with chronic diseases at family medicine clinics in National Guard Health Affairs, Riyadh, Saudi Arabia, from November 2012 until May 2013.

Consecutive patients who visited the attended clinic and are known to have chronic disease, who are on medications for more than one year, who are not known to have cognitive impairment or psychiatric illness, and aged 18 years or older were considered initially eligible for the study. Patients who meet the above inclusion criteria were approached at the end of the consultation and were asked to participate in the study. After obtaining the informed consent, patients were interviewed to collect demographic data, whether they are helped by others in taking medications or not, if having mental disorder not recorded in the chart, and Mini-cog test was done to all patients aged 65 years or more [20]. Exclusion criteria include inability to understand spoken words due to hearing loss, severe visual impairment hindering reading, inability to pass Mini-cog test, being assessed in taking medication by family members, or having psychiatric illness not written in the file. Later, the final eligible patients were asked to fill the questionnaires addressing medications beliefs, adherence, and their perception about the adequacy of the information they have received about their medications. If the patients were having difficulty in reading or understanding they were assisted by the interviewer and care was taken not to be judgmental during the interview in order to avoid social desirability bias.

Self-reported adherence was assessed using Morisky Medication Adherence Scale (MMAS-8). This scale has been validated on a sample of 1367 hypertensive patients with Cronbach alpha of 0.83 and has been used in many languages [21–23]. It consists of eight items that address specific medication taking behavior and adherence both intentional and unintentional. The first seven items have dichotomous responses (yes/no) and they are formulated in a way to avoid acquiescence bias, whereas the eighth item has 5 point Likert scale response. The total score of MMAS-8 ranges from 0 to 8 with higher scores representing higher adherence. Those who score less than 6 were considered to have low adherence and those who scored 6 or more were considered as high adherers [24].

Beliefs about medicines questionnaire (BMQ) was used to assess patients' beliefs about medications [25]. BMQ is a tool to measure cognitive representation of medication and has been shown to be valid and reliable in variety of health conditions. It has two parts, one assessing patients' beliefs about their own medications (BMQ-specific) and the other assessing patients' beliefs about medications in general (BMQ-general). The BMQ-specific part covers two themes; specific necessity theme evaluates patients' view about the necessity and importance of their medication, whilst specific concern theme comprises patients' beliefs about potential harm and adverse effects of their own medications and each of which has a score ranging from 5 to 25. A high score in necessity theme means that patients think their medications are important to them; on the other hand a high score in the concerns theme means that patients are concerned and worried about their own medications. Likewise, BMQ-general part has two themes; general overuse theme assesses how patients perceive the extent of medication usage, and general harm theme represents patients' beliefs about harmful nature of medication in general. The scores of the last two themes range from 4 to 20, and high score in each theme means negative perception about medications in general.

The extent of medication information given to the patients was evaluated by patients' views about adequacy of medication information. This was measured using patients' perceived adequacy of medications information scale which has items that were adopted from satisfaction with information about medicine scale [26]. The used scale consists of 15-items and two subscales. The first 9-items comprise action and usage subscale which assess patients views about the adequacy of the information they received regarding name of medication, reasons for taking medication, for how long to use it, how to use it, what it does, how it works, how to tell if it is working, how long it will take to act, and how to take further supply. The last 6-items make the potential problems with medications subscale and assess the information received regarding potential side effects, chances of having side effects in patient, what to do if side effect occurs, what to do if patient missed a dose, whether medication interacts with others, and whether medication causes drowsiness. For each item, participants who respond that they have received enough or more than enough information will get a score of one, while participants who rate the amount of information as little or none received will get a zero score. The overall score ranges from 0 to 15; the higher the score in each subscale, the more the perceived adequacy of medication information.

Arabic translation and linguistic validation for MMAS-8 were done by an international institute for linguistic validation, MAPI Institute, and it was provided by the original author. In addition, a forward translation from English to Arabic for BMQ was done by two independent native Arabic linguists, and after reconciliation and involving an expert in behavioral medicine an initial version was developed. The same was done for the perceived adequacy of medication information which was adopted from satisfaction with information about medicine scale and the international guidelines were followed in all translation processes [27]. Cognitive debriefing of the survey was done on 20 patients from our clinic and they were not included in this study. Few corrections were made to improve clarity of questions and to avoid leading questions and the final version has been shown to have good face validity. The literal back translation of the final versions was done by a native speaker of original language and they were approved by the original authors.

Based on previous data the estimated average proportion of low adherence was 52% [28, 29], and using NCSS-PASS (Power Analysis and Sample Size) 11 V11.0.7 program with desired 95% confidence interval of ±5, the estimated required sample size was 401.

Data management and analyses were carried out using PSAW (Predictive Analytic Software) V18 and a priori *α* of 5% was set for all analysis. Descriptive statistics was used in describing patients' characteristics and their degree of adherence, Chi-square test, Mann-Whitney *U* test, and Student *t*-test were used where appropriate. Pearson's correlation was used to assess bivariate association between adherence score, beliefs about medicines score, and information adequacy. Additionally, associations between adherence, beliefs, and perceived adequacy of medication information were evaluated after accounting for other variables and predictors using multiple linear regression. Moreover, Cronbach's alpha used to test internal consistency and reliability of the questionnaires and validity was evaluated using known-group validity method by assessing correlations between the questionnaires, and it was assumed that there is positive relationship between adherence degree, positive beliefs, and high adequacy of medication information.

The research protocol was evaluated and approved by institutional review board and an informed consent was obtained from all subjects.

## 3. Results

**Figure 1:**
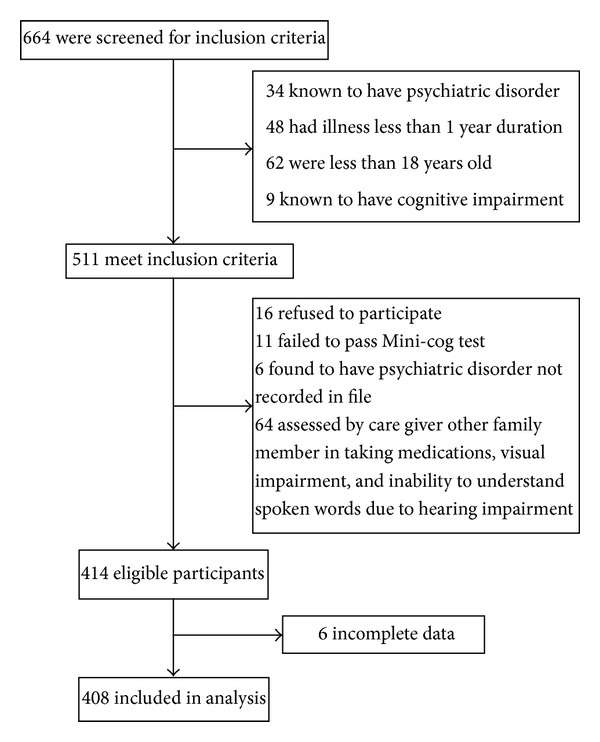
Flowchart indicating the selection process of study participants.

Among the consecutive patients who were screened during the study period, 414 were found to be eligible for the study (Figure 1). Of them, 408 participants were included in the final analysis and their characteristics are shown in Table 1. The participants were on average 51 years old, almost half of them were female, and more than the half were illiterate or barely finished few years of primary school. Furthermore, 72.5% of the participants had 2 or more medical conditions with average illness duration of more than 7 years, and the majority had 4 or more medications. Diabetes was the most common disease among the participants followed by hypertension.

Overall 56.9% of participants were found to have low adherence (Table 1). Those who reported low adherence tend to have statistically significantly younger age, higher literacy level, higher number of medication, and shorter illness duration when compared to high adherence group. Majority (71.1%) of the study participants were diabetics and 51% (148 out of 290) of them reported low adherence. The second most common chronic disease among the participants was hypertension (54.7%) and around 50% (113 out of 223) of them were found to have low adherence. The highest reported low adherence (72%) was among patients with asthma (42 out of 58). Moreover, both low and high adherence group scored high in the necessity beliefs, though the score was significantly higher in the high adherence group. Participants with low adherence had significantly higher level of concerns about their medications and stronger beliefs that medications are overused, harmful, and addictive in nature. In addition, low adherers view that the information they have received regarding their medications is less adequate when compared to patients with high adherence (Table 1). Participants' reported adherence behavior is shown in Table 2.

Using Pearson's bivariate correlation it was found that there was positive association between adherence score (using MMAS-8) and BMQ specific necessity (*r* = 0.53; *P* < 0.001) and information adequacy (*r* = 0.57; *P* < 0.001). Conversely, there was negative association between adherence score and BMQ specific concerns (*r* = −0.67; *P* < 0.001), general overuse (*r* = −0.47; *P* < 0.001), and general harm (*r* = −0.66; *P* < 0.001). Moreover, when conducting multiple linear regression after adjusting for other covariables, it was found that age, number of medical conditions, BMQ specific necessity beliefs, and information adequacy were positively associated with adherence score while number of medications and BMQ specific concern and general harm were negatively associated with adherence score (Table 3). Furthermore, after adjustment, BMQ-general overuse beliefs were no longer associated with adherence (Table 3). The used regression model explains 78% of variance in the adherence, while specific beliefs explained 27.7% of the variance and perceived information adequacy explained 32.3% of the variance in adherence.

Pearson's coefficients of correlation between perceived adequacy of medication information and BMQ scores were *r* = 0.28, −0.39, −0.34, and −0.40 (*P* < 0.001) for specific necessity, specific concerns, general overuse, and general harm, respectively, and Table 4 shows the multivariable linear association between beliefs and information adequacy subscales after accounting for other covariables.

Reliability testing for MMAS-8 showed Cronbach's *α* of 0.795 and for the perceived adequacy of medication information questionnaire Cronbach's *α* was 0.794.

## 4. Discussion

This study which was conducted in family medicine clinics found that the majority of patients with chronic disease reported low adherence and it was associated with negative beliefs about medications and the perception of medication information inadequacy. The used measurement tools were found to be reliable with Cronbach's alpha >0.7 and valid using a known-group method.

The high number of reported low adherence was similar to another study conducted on diabetics in the Al Hasa district in Saudi Arabia which found nonadherence to mediation to be 57.5% [28]; however the reported low adherence among diabetic patients in this study was 51%. Of the hypertensive patients in the current study 50% were found to have low adherence and it was near to a previous report, albeit different definition was used, of 47% prevalence of nonadherence among hypertensive patients using pill counts method [29]. Moreover, the reported low adherence among sufferers of chronic diseases was much higher than another finding from a recent study which measured adherence intension using a nonvalidated modified version of the MMAS-4 [30]. The investigators of this study found that 40% of the patients had low and variable intension to adhere to their medication, yet nonadherence can be intentional and unintentional and both of which were assessed in our study using MMAS-8. The differences between this study findings and other studies can be explained by the different methodologies used, type of the patients, setting of the study, and illnesses severity.

Despite cultural differences, beliefs about medications influence adherence among our patients in Saudi Arabia. In this study there was bivariant association between adherence and beliefs about specific necessity, specific concerns, general overuse, and general harm. However, after using multivariable analysis medication adherence was associated with specific beliefs about medications (necessity and concerns) and general beliefs about the harmful and addictive nature of medications but not with general overuse beliefs and these findings were near to other reports [9, 31]. Furthermore, specific beliefs explain 27.7% of the variance in adherence and despite that there were differences in the specific necessity beliefs between high and low adherers, both of the two groups think that their medications are necessary to them and this finding was similar to other findings [12].

The current study highlights the importance of education about medication in order to improve adherence. In the present study, there was a direct association between patients' views about adequacy of information they had received about their medications and their degree of adherence. In addition, there was an indirect relationship between adherence and medication information, as the extent of medication information given to patients influences their beliefs which in turn affect adherence. Khan et al. [28] found a relationship between adherence and the extent of information received using four items and this study supports this finding using fifteen items addressing most of information needed by patients. Indeed, the more the information patients received about action and usage of their medications the more the patients view their medications necessary; also the less the amount of information the patients received about the potential problems and side effects about their medications the more concerned the patients were. On the contrary, other investigators found that some of the patients who were educated about the side effects of medication became more concerned and their risk perception increased [17]. This finding is true if the medications have potential side effects and risks like benzodiazepines as in the previous study; however the findings in the current study should be interpreted and viewed in the light of medical conditions and medication included. Interestingly, patients who perceived medications as harmful and addictive in nature felt that all types of medications information they had received were inadequate and this could be due to the negative beliefs and concerns about the unfamiliar medications.

Other investigators found that males tend to have lower adherence when compared to females [28, 30]. In this study males also reported lower adherence than females but this was not statistically significant. The high number of patients with low literacy level was near to other findings [28, 30]. This finding is expected because our study participants are sufferer of chronic diseases and they tend to be of older age. Moreover, low adherence was reported more among patients with higher level of education. This could be due to the effect of social desirability bias as those with lower literacy level were helped more by interviewer and they were more likely to report high adherence; another possible reason is that patients with low literacy level may be unaware about potential problems and side effects of medications. Nevertheless, after adjustment, literacy level was not found to be significantly associated with adherence. Similarly, other investigators found that literacy level did not influence adherence [10, 25, 27]. Majority of asthmatic patients reported low adherence. This could be due to more negative beliefs held by asthmatic patients about their medication than other patients because after accounting for beliefs it was not found that being asthmatic is significantly associated with low adherence. Other investigators who reported asthma as predictor for low motivation for adherence did not adjust for patients' beliefs and the same was true for other included chronic diseases [30]. Furthermore, as expected from many previous studies, as the number of medications increases the adherence decreases [9, 30]. However patients with multiple comorbidities who tend to have high number of medication and more complex regimen were found to have higher tendency to adhere to their medications. This finding can be explained by illness perception as those with multiple diseases may perceive their condition as more severe than others and this factor was not included in the multivariable analysis.

A self-report method was used to assess adherence in this study; nonetheless it is considered to be an effective method and those who report poor adherence are most likely truthful [1]. Up to our knowledge, this is the first study that measured adherence using a validated tool in the Middle East and nobody has examined relationship between adherence using MMAS-8 and beliefs using BMQ and patients' perception of medication information adequacy. Self-efficacy was not included in this study; however patients who were assisted in taking their medication were considered noneligible. In addition, strict inclusion criteria were applied and response bias was not a problem in this study. Care was taken to be nonjudgmental during interview, yet interviewer bias cannot be ruled out but it is believed to have a minimal impact on study validity. Finally, care should be taken about generalizability of this study findings because it was conducted on patients who had mostly uncomplicated asymptomatic illnesses and most of the patients' medications had benign side effect profile.

Further studies are needed to examine the effect illness perception and illness knowledge on adherence as well as intervention studies to assess the impact of tailored educational interventions addressing patients' negative beliefs on the degree of adherence.

## 5. Conclusion

The prevalence of low adherence among patients with chronic diseases in family medicine clinics is alarming and it is related in part to negative beliefs about medications and insufficiency of information provided to patients regarding their medications. Discussing medications adherence and beliefs with the patients is important and should be a part of the usual clinical care of chronic diseases as improving medicine taking behavior may have a far greater impact on clinical outcomes than improvement in treatment [6].

## Figures and Tables

**Table 1:** Characteristics of study participants and their level of adherence.

Characteristics	Total *N* = 408	Low adherence *N* = 232 (56.9%)	High adherence *N* = 176 (43.1%)
Age*			
Mean (±SD)	51.2 ± 13.3	48.3 ± 14.2	54.9 ± 11.1
Gender *N* (%)^a^			
Male	207 (50.7)	125 (53.9)	82 (46.6)
Female	201 (49.3)	107 (46.1)	94 (53.4)
Literacy level *N* (%)*			
Less than primary school	214 (52.5)	93 (40.1)	121 (68.8)
School degree	142 (34.8)	102 (44)	40 (22.7)
Higher education	52 (12.7)	37 (15.9)	15 (8.5)
Number of medical conditions*			
Mean (±SD)	2.28 ± 1.2	2.15 ± 1.2	2.46 ± 1.1
Average duration of illness*			
Mean (±SD)	7.64 ± 5.9	8.87 ± 6.8	6.01 ± 4.1
Number of medications*			
Mean (±SD)	4.42 ± 2.6	4.69 ± 3.1	4.06 ± 1.8
Diabetes mellitus *N* (%)*	290 (71.1)	148 (63.8)	142 (80.7)
Hypertension *N* (%)*	223 (54.7)	113 (48.7)	110 (62.5)
Hyperlipidemia *N* (%)	210 (51.5)	122 (52.6)	88 (50)
Asthma *N* (%)*	58 (14.2)	42 (18.1)	16 (9.1)
Hypothyroidism *N* (%)	52 (12.7)	25 (10.8)	27 (15.3)
BMQ-specific necessity score^b^			
Mean (±SD)*	17.9 ± 2.8	17.0 ± 2.9	19.2 ± 2.1
BMQ-specific concerns score^b^			
Mean (±SD)*	15.7 ± 3.6	17.6 ± 2.9	13.2 ± 2.3
BMQ-general overuse score^b^			
Mean (±SD)*	13.6 ± 2.3	14.5 ± 2.1	12.4 ± 2.0
BMQ-general harm score^b^			
Mean (±SD)*	10.4 ± 2.8	11.8 ± 2.5	8.5 ± 2.0
Perceived adequacy of medication information^c^			
Mean (±SD)*	7.9 ± 3.3	6.2 ± 2.8	10.2 ± 2.5

*Statistical significant differences between groups.

^a^Percentages are in columns orientation within the total number of participants and adherence categories.

^b^BMQ: beliefs about medicines questionnaire. Specific-necessity and concerns scores possible range from 5 to 25. General overuse and harm scores possible range from 4 to 20.

^c^Perceived adequacy of medication information has possible scores range from 0 to 15.

**Table 2:** Participants' reported adherence behavior.

Self-reported adherence as measured by MMAS-8	
Item	Percentages of patients who answered yes
Do you sometimes forget to take your medicine?	63%
People sometimes miss taking their medications for reasons other than forgetting. Thinking over the past two weeks, were there any days when you did not take your medicine?	42.9%
Have you ever cut back or stopped taking your medication without telling your doctor, because you felt worse when you took it?	40.7%
When you travel or leave home, do you sometimes forget to bring along your medication?	38.7%
Did you take your medicine yesterday?	80.4%
When you feel like your health condition is under control, do you sometimes stop taking your medicine?	33.1%
Taking medication everyday is a real inconvenience for some people. Do you ever feel hassled about sticking to your treatment plan?	46.8%
How often do you have difficulty remembering to take all your medications?	
(i) Never/rarely	27%
(ii) Once in a while	34.3%
(iii) Sometimes	32.6%
(iv) Usually	5.6%
(v) All the time	0.5%

**Table 3:** Association between adherence score with beliefs about medications and other significant variables in the regression analysis^a^.

Variables	*r* (95% CI)	*β* (95% CI)
Age	0.31 (0.22–0.41)***	0.12 (0.06–0.18)***
Number of medical conditions	0.14 (0.04–0.24)**	0.13 (0.02–0.24)**
Number of medications	−0.14 (−0.24–−0.04)**	−0.20 (−0.29–−0.11)***
BMQ-specific necessity	0.53 (0.44–0.62)***	0.30 (0.26–0.34)***
BMQ-specific concerns	−0.67 (−0.74–−0.6)***	−0.40 (−0.47–−0.33)***
BMQ-general overuse	−0.47 (−0.55–−0.39)***	0.03 (−0.05–0.11)
BMQ-general harm	−0.66 (−0.74–−0.58)***	−0.24 (−0.3–−0.18)***
Perceived adequacy of medication information	0.57 (0.49–0.65)***	0.18 (0.13–0.23)***

^a^Adjustment was done for age, gender, literacy level, illness duration, number of medical illnesses, comorbidities, number of medications, beliefs, and perceived medication information adequacy. Only significant variables found in the regression analysis in addition to beliefs are presented in this table.

Note: in the used linear regression model Intercept = 5.387; *R* ^2^ = 0.78;  *F* = 89;  *P* < 0.001; df = 16; *r*: Pearson's bivariate correlation coefficient; *β*: multiple linear regression coefficient; CI: confidence interval; BMQ: beliefs about medicines questionnaire; *P < 0.05; **P < 0.01; ***P < 0.001.

**Table 4:** Summary of regression analyses for association between beliefs and information adequacy subscales^a^.

Perceived adequacy of medication information subscales	BMQ-specific necessity *β* (95% CI)	BMQ-specific concerns *β* (95% CI)	BMQ-general overuse *β* (95% CI)	BMQ-general harm *β* (95% CI)
Action and usage	0.26 (0.15–0.37)***	−0.06 (−0.15–0.03)	−0.24 (−0.34–−0.14)***	−0.19 (−0.30–−0.08)***
Potential problems of medication	−0.06 (−0.17–0.05)	−0.21 (−0.31–−0.11)***	−0.02 (−0.13–0.09)	−0.15 (−0.26–−0.04)**

^a^Adjusted for age, gender, literacy level, number of medical conditions, number of medications, and illnesses duration; BMQ: beliefs about medicines questionnaire; *β*: multiple linear regression coefficient; CI: confidence interval; *P < 0.05;  **P < 0.01;  ***P < 0.001.
